# On Excision of Joints for Disease, and Especially of the Knee, Hip, and Elbow

**Published:** 1870-11

**Authors:** 


On Excision of Joints for Disease, and Especially of the Knee, Hip, and Elbow.—At a recent meeting of the Royal Medical and Chirurgical Society, Mr. F. J. Grant read a paper on this subject, in which he endeavored to set forth the conditions of disease which indicate excision of the joints in general. He has given the analysis of a series of cases, in regard to the Pathology, the operation, and its results.

The necessity for either excision or amputation in joint diseases, implies incurability by non-operative treatment. As regards excision, incurability is defined as that condition in which the joint has become *functionally useless* by destruction of the articular cartilages, without anchylosis, but while the *constitution* still retains the reserve power requisite for the long process of reparative union—averaging three months, after the removal of the diseased bone. Cases which do not have this requisite constitutional reserve power, but which are accompanied by prolonged hectic and exhaustion, will be so far unfavorable for excision, and must be submitted to the alternative of amputation.

As compared with the *natural cure*, by anchylosis, a possible result in joint disease, excision is preferably in proportion to the very long recovery by the natural process, a probation which entails long-continued suffering, and leaves the system inadequate to the contingencies of after life.

The author gave, at length, histories of the illustrative cases and their analyses. The following is a brief abstract:

1. *Excision* proved successful, by one operation in 16 out of 20 cases of the joints referred to.

2. Of the 4 unsuccessful cases, by this one operation, 3 were of *scrofulous* disease, and of the knee-joint, out of 9 cases; the remaining one being of *chronic* synovitis, and of the elbow-joint, out of 5 cases.

3. *Re-excision* was resorted to in 2 of the 4 cases; 1 knee-joint and one elbow-joint, the latter with a successful result.

4. *Secondary amputation* in three of the four cases. All three were knee-joint cases, and of scrofulous disease; one of which had been subjected to re-excision. The three amputations made *rapid recoveries*. These results tend to show that, if the attempt to preserve a limb by excision, and even by re-excision of a large joint, as the knee, should fail, the operation is not unfavorable to secondary amputation to save life.

5. *No death* ensued in any of the 20 cases of knee, hip, or elbow joints, whatever had been the condition of disease, or the operation, —excision, re-excision, or secondary amputation.—*Lancet.*

